# Genetic parameters of resistance to pasteurellosis using novel response traits in rabbits

**DOI:** 10.1186/s12711-020-00552-8

**Published:** 2020-06-26

**Authors:** Merina Shrestha, Hervé Garreau, Elodie Balmisse, Bertrand Bed’hom, Ingrid David, Edouard Guitton, Emmanuelle Helloin, Guillaume Lenoir, Mickaël Maupin, Raphaël Robert, Frédéric Lantier, Mélanie Gunia

**Affiliations:** 1 GenPhySE, INRAE, ENVT, Université de Toulouse, 31326 Castanet-Tolosan, France; 2 PECTOUL, INRAE, 31326 Castanet-Tolosan, France; 3 GABI, INRAE, AgroParisTech, Université Paris-Saclay, 78352 Jouy-en-Josas, France; 4 PFIE, INRAE, 37380 Nouzilly, France; 5 ISP, INRAE, Université François Rabelais de Tours, UMR 1282, 37380 Nouzilly, France; 6 HYCOLE, Route de Villers-Plouich, 59159 Marcoing, France; 7 HYPHARM SAS, La Corbière, Roussay, 49450 Sèvremoine, France; 8 EUROLAP, Le Germillan, B.P. 21, 35140 Gosné, France

## Abstract

**Background:**

Pasteurellosis (*Pasteurella* infection) is one of the most common bacterial infections in rabbits on commercial farms and in laboratory facilities. Curative treatments using antibiotics are only partly efficient, with frequent relapses. Breeding rabbits for improved genetic resistance to pasteurellosis is a sustainable alternative approach. In this study, we infected 964 crossbred rabbits from six sire lines experimentally with *Pasteurella multocida.* After post-mortem examination and bacteriological analyses, abscess, bacteria, and resistance scores were derived for each rabbit based on the extent of lesions and bacterial dissemination in the body. This is the first study to use such an experimental design and response traits to measure resistance to pasteurellosis in a rabbit population. We investigated the genetic variation of these traits in order to identify potential selection criteria. We also estimated genetic correlations of resistance to pasteurellosis in the experimental population with traits that are under selection in the breeding populations (number of kits born alive and weaning weight).

**Results:**

Heritability estimates for the novel response traits, abscess, bacteria, and resistance scores, ranged from 0.08 (± 0.05) to 0.16 (± 0.06). The resistance score showed very strong negative genetic correlation estimates with abscess (− 0.99 ± 0.05) and bacteria scores (− 0.98 ± 0.07). A very high positive genetic correlation of 0.99 ± 0.16 was estimated between abscess and bacteria scores. Estimates of genetic correlations of the resistance score with average daily gain traits for the first and second week after inoculation were 0.98 (± 0.06) and 0.70 (± 0.14), respectively. Estimates of genetic correlations of the disease-related traits with average daily gain pre-inoculation were favorable but with high standard errors. Estimates of genetic and phenotypic correlations of the disease-related traits with commercial selection traits were not significantly different from zero.

**Conclusions:**

Disease response traits are heritable and are highly correlated with each other, but do not show any significant genetic correlations with commercial selection traits. Thus, the prevalence of pasteurellosis could be decreased by selecting more resistant rabbits on any one of the disease response traits with a limited impact on the selection traits, which would allow implementation of a breeding program to improve resistance to pasteurellosis in rabbits.

## Background

*Pasteurella multocida*, a gram-negative bacterium, affects various birds and mammals worldwide, including humans [1]. It is an opportunistic pathogen that normally resides as part of the normal microbiota in oral, nasopharyngeal, and upper respiratory tracts in mammals, birds, and other species [2]. Infection with *P. multocida* (pasteurellosis) causes a variety of clinical manifestations in various species, including fowl cholera in poultry, atrophic rhinitis in pigs, and hemorrhagic septicemia in cattle and buffalo [2].

In rabbits, rhinitis (‘snuffles’), pneumonia, septicemia, abscesses, and mastitis are some of the clinical manifestations caused by different strains of *P. multocida* [3]. Pasteurellosis is one of the most common bacterial infections both on commercial farms and in laboratory rabbits [3]. It is a highly epizootic infection and an economically major disease in rabbit meat industry. Eady et al. [4] reported a mortality rate of ~ 50% in a grower rabbit population that was diagnosed with bacterial infection (predominantly *Staphylococcus aureus* and *P. multocida*). Lopez et al. [5] reported pasteurellosis as the first cause of culling of young rabbit does.

Vaccinations and curative treatments using antibiotics against pasteurellosis are only partly efficient, and relapses of pasteurellosis are frequent [6]. Thus, prophylactic measures are considered as economically efficient. In the rabbit meat breeding industry, antibiotics are used as a prophylactic measure, together with proper ventilation and strict environmental hygiene in housing buildings, to prevent and control the spread of infection. However, the use of antibiotics has several disadvantages. One risk is the development of antibiotic-resistant bacteria, which could spread to other species, including humans. In particular, the development of antibiotic-resistant bacteria has been observed in intensive farms [6]. Ferreira et al. [7] reported that 22 of 46 strains of *P. multocida* isolated from rabbits in Brazil showed resistance to at least one type of antibiotic. Wilson and Ho [2] identified multiple antibiotic-resistant genes in different strains of *Pasteurella* species. Another adverse effect of the use of antibiotics is dysbiosis, which is an imbalance of the normal microbiota of an organism that can occur when antibiotics are added to the feed [8]. The use of antibiotics can also mask the disease, thus preventing any selective advantage of natural resistance against pasteurellosis in animals [9]. Finally, the use of antibiotics is not well accepted by consumers [10] and various policies have been implemented to reduce exposure of humans and animals to antibiotics. Thus, the development of a sustainable alternative approach is desirable to reduce the use of antibiotics, while maintaining a low prevalence of pasteurellosis. One approach could be to breed rabbits for improved genetic resistance to pasteurellosis, which would have a lower probability of developing the disease. Moreover, the presence of resistant rabbits in a population will decrease disease transmission, thus reducing the risk of infection for susceptible individuals, imparting a certain level of herd immunity.

Previous studies have suggested the possibility of selection for resistance to pasteurellosis in rabbits, since low to moderate heritability estimates ranging from 0.03 (± 0.01) to 0.28 (± 0.16) were found [11–13]. Most of these studies were based on observable clinical signs of infection from field data. Such field studies have three drawbacks that can lead to inaccurate diagnosis of pasteurellosis: (1) not all infected rabbits show visual signs of infection but remain carriers of the bacteria; (2) some clinical signs such as rhinitis and pneumonia in rabbits are also observed after infection with other bacteria such as *Staphylococcus* or *Streptococcus* [3]; and (3) due to an uneven exposure to infection in the field, a rabbit may not show its true response potential. Such inaccurate diagnosis of the health status can reduce estimates of heritability [14]. These drawbacks can be avoided by analyzing response traits in a population that is experimentally exposed to the pathogen.

The aim of our study was to identify criteria to genetically select rabbits for increased resistance to pasteurellosis. We estimated the genetic parameters for pasteurellosis resistance traits in a population that was experimentally infected with a strain of *P. multocida*. We also estimated the genetic correlations of resistance traits with the production traits that are being selected for in commercial rabbits.

## Methods

### Populations

We used two datasets. The first dataset contained information on 11,971 purebred rabbits born in 2016 and 2017 from six maternal lines: two lines from each of the following breeding companies Eurolap, Hycole, and Hypharm (Table 1). These lines have been operated as closed populations since their establishment between 1980 and 1985 (except for one line established in 1997). In these lines, maternal line traits, such as prolificacy, fertility, functional longevity, number of teats, direct and maternal effects on weaning weight, and homogeneity of weight at birth are continuously improved. For this study, we considered only two major traits that are under selection across all lines, i.e. number of kits born alive (NBA) and weaning weight (WW). The second dataset included data from an experimental population of crossbred rabbits that were submitted to an experimental infection trial with *P. multocida*. The experimental rabbit population included 1030 crossbred rabbits. These crossbreds were progenies from six sire lines (two lines from each of the breeding companies Eurolap, Hycole, and Hypharm) bred to one dam line (INRA 1777) (Table 2) that has been selected for traits such as number of kits born alive per litter, and direct and maternal effects on weaning weight. Hence, this experimental population was representative of the commercial breeding dams used by French rabbit breeders.

**Table 1:** Description of the commercial population of 11,971 purebred rabbits

Line	Number of sires	Number of dams	Number of purebreds
1	11	199	2433
2	32	312	2231
3	32	235	2121
4	19	149	1789
5	12	378	2786
6	10	204	611

**Table 2:** Description of the experimental population of 1030 crossbred rabbits

Sire line	Number of sires	Number of dams (line 1777)	Number of crossbreds
1	11	48	157
2	10	45	169
3	10	48	155
4	12	54	167
5	11	48	188
6	11	61	194

The experimental population was generated in 2016 at the Pôle Expérimental Cunicole de Toulouse (INRAE PECTOUL) experimental unit by artificial insemination across five reproduction batches, at intervals of 1–3 months. The experimental population (1030 crossbred rabbits) was produced by mating 65 sires with 112 dams. The same sires and dams were used for the whole experiment. Breeding companies and INRAE provided pedigree information for the sires and dams, respectively. Each sire line contributed on average 171.7 progenies (Table 1), while each sire contributed on average 15.6 crossbred progenies (from 2 to 23), and each dam on average 9.2 crossbred progenies (from 1 to 21). The crossbred rabbits were delivered in a span of 3 consecutive days for the first batch, and of 4 consecutive days for the other batches. At weaning, kits without visible disease syndromes were pre-sorted for the experiment. Then, rabbits were chosen to achieve a balanced distribution of gender and paternal and maternal origins. The first batch included 110 rabbits and the remaining four batches included 230 rabbits, each. This population was raised in a new building with a high level of biosecurity and was monitored for the usual rabbit pathogens.

The 65 sires that produced the experimental population were also used to produce the purebred progenies in the selection population of the breeding companies, along with other related sires. The selection population (11,971 purebred rabbits) was obtained by mating 116 sires with 1477 dams (Table 2). Each sire contributed on average 75.6 purebred progenies (from 1 to 290), and each dam on average 6.3 purebred progenies (from 1 to 28). The pedigree contained 20,206 rabbits across seven generations and phenotypic information was available for 12,951 of these rabbits (11,971 purebreds and 980 crossbreds) in the data file. Of the 11,971 purebred rabbits from the selection population, 1705 had records on both NBA and WW, 548 on NBA only, and 9718 on WW only.

### Inoculation of the crossbred experimental population

In total, 50 rabbits (10 rabbits per batch) were used as controls and 980 rabbits were inoculated with *P. multocida*. The 1030 rabbits were transported in cages (5 rabbits per transport cage) to the Plateforme d’Infectiologie Expérimentale (INRAE PFIE) at 36 days of age, 1 day after weaning. At PFIE, they were all housed in cages identical to each other (with the same 5 rabbits per cage as during transport) in two separate rooms. Control rabbits were housed in the same rooms as the inoculated rabbits. Before inoculation, 16 of the 980 rabbits died or were euthanized due to digestive disorders, mostly epizootic rabbit enteropathy, which is a potentially fatal gastrointestinal disease with a currently unknown etiology but with a strong bacterial hypothesis [15].

**Fig. 1:**
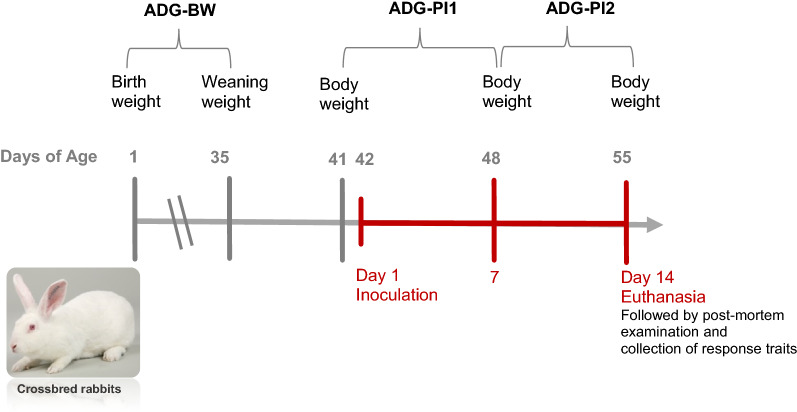
Overview of the experimental infection trials of crossbred rabbits, with age at body weight measurement, inoculation, and euthanasia. ADG-BW: average daily weight gain calculated from birth to weaning pre-inoculation; ADG-PI1: average daily weight gain calculated during first week post-inoculation; ADG-PI2: average daily weight gain calculated during second week post-inoculation. The red line refers to the stage post-inoculation, from the first day of inoculation to the last day of the experiment when rabbits were euthanized

At PFIE, the experimental population was left to adjust to the new environment for a week and inoculations were performed at 42 days of age by injecting the 964 rabbits subcutaneously between the shoulder blades with a standardized dose of 8000 bacteria in 0.1 mL saline solution, of the pyrogenic strain CIRMBP-0884 of *P. multocida* from a stock that is kept frozen at − 80 °C and checked for concentration at each inoculation. This strain was chosen based on a previous study [16] in which 174 *Pasteurella* strains isolated from French rabbits were characterized phenotypically and with molecular parameters. All the strains were genotyped by MLVA [16], and for 20 of the 174 strains that were selected to represent their diversity, pathogenicity was tested in rabbits by using a standard dose. The median lethal dose was not determined. Based on this information, the CIRMBP-0884 strain of *P. multocida* (also known as the LVT62 strain) [17], conserved at the Centre International de Ressources Microbiennes—Bactéries Pathogènes (CIRM-BP), was found to be a virulent strain that belongs to one of the major groups of *Pasteurella* field isolates. Resistance to pasteurellosis in rabbits should take resistance to different strains of *P. multocida* into account. Although the nasal route is the major natural route of penetration for *Pasteurella* [3], we used subcutaneous injection because Helloin et al. [16] found that it showed better reproducibility and quantifiable infection compared to the intranasal route. Health status of the rabbits was monitored daily for 14 days post-inoculation. Critically ill rabbits were euthanized for welfare reasons. All the other rabbits were euthanized at 14 days post-inoculation and their bodies were examined for signs of pasteurellosis. A brief timeline of this experiment is shown in Fig. 1. Of the 964 inoculated crossbred rabbits, 844 remained alive until the end of the experiment and were euthanized on day 14 post-inoculation. Among the 120 rabbits that died or were euthanized prior to the end of the experiment, 109 rabbits were confirmed to have died of pasteurellosis, thus the data on these were included in the analysis. In total, 953 crossbred rabbits were used for analysis.

### Traits

Both commercial selection traits, WW and NBA, were recorded in the six purebred populations by the breeding companies. Rabbits were weaned between 27 and 36 days of age, depending on the line.

In the crossbred experimental population, the following disease-related and performance traits were recorded:

#### Abscess

During post-mortem examination, the same two scientists recorded the occurrence of abscesses during the experiment, by using a scoring grid. Presence or absence of abscesses was recorded and scored from 0 to 4, on different parts of the body: inoculation site, head, neck, chest, back, forelegs, hind legs, ribcages, abdomen, rump, thoracic cavity, peritoneal cavity, pleura, heart, lungs, liver, spleen, kidney, stomach, and digestive tract. Furthermore, a single score from 0 to 4, was assigned to each rabbit depending of the extent of abscess dissemination on different parts of the body. Details on the scores are in Table 3.

**Table 3:** Description of scores for disease-related traits: abscess and bacteria

Score	Abscess	Bacteria
0	No signs of abscess	No growth of *Pasteurella* in the culture from the tissue samples
1	Lesions observed at inoculation site	Growth of *Pasteurella* in the culture from abscess only
2	Lesions also observed on front part of the body	Growth of *Pasteurella* in the culture from the tissue samples of one organ
3	Lesions also disseminated on rear part of the body (abdomen, rear legs)	Growth of *Pasteurella* in the culture from the tissue samples of two organs
4	Lesions also observed inside organs and cavities	Growth of *Pasteurella* in the culture from the tissue samples of all organs

#### Bacteria

Post-euthanasia, tissue samples from the spleen, lung, liver, and abscesses (from any area) were collected, rapidly frozen and kept at − 80 °C (2 to 3 months) until they were transferred to the Laboratoire de Touraine (Tours, France) and then homogenized individually for culture to identify and quantify *P. multocida*. Liver tissue was sampled only from rabbits that died or were euthanized prior to the end of the experiment because rabbits that die at an early stage may not show any abscess and sampling liver tissue increases the chance of obtaining bacterial cultures of *P. multocida*.

In the laboratory, the cultures from lung, spleen, and liver samples were scored for the presence of *P. multocida* by enumeration of bacteria (viable plate count), while the culture obtained from abscess samples was only checked to determine if it belonged to the *P. multocida* species. The bacterial counts for each tissue were then rescored as 0 (no growth), 1 (numerable colonies), and 2 (innumerable colonies), which were used to generate a final score from 0 to 4 for each rabbit, as described in Table 3.

#### Resistance

**Fig. 2:**
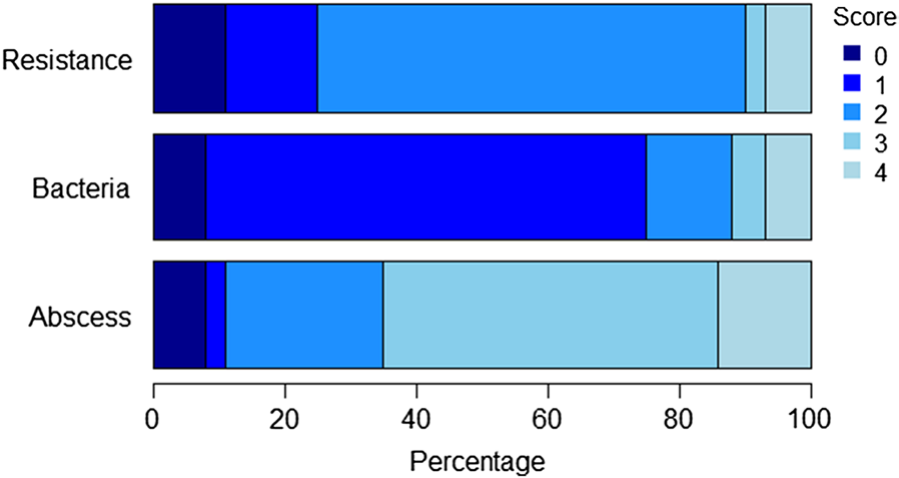
Percentage of rabbits with different scores for each disease-related response trait

For each rabbit, a score for resistance to pasteurellosis from 0 to 4 was derived by combining the scores for extent of abscesses (0 to 4), extent of bacteria growth from the tissue samples (0 to 4), and the status (dead/alive) of the rabbits at the end of the experiment. The description of the scores is in Table 4. The distribution of rabbits across scores of disease-related traits is in Fig. 2.

**Table 4:** Description of scores for the disease-related trait resistance

Score	Resistance
0	Presence of at least signs of abscesses or bacteria irrespective of severity score
	Dead from pasteurellosis during the experiment
1	Abscess score: 4
	Growth of bacteria in the culture
	Alive until the end of the experiment
2	Abscess score: 2 or 3
	Bacteria score: 0 or 1
	Alive until the end of the experiment
3	Abscess score: 1
	Alive until the end of the experiment
4	No signs of abscess
	No growth of bacteria in the culture
	Alive until the end of experiment

#### Growth traits

Body weight was measured at birth, at weaning, 1 day before inoculation, and on days 7 and 14 post-inoculation. Average daily weight gain [ADG (g/days)] was calculated by dividing the difference in body weight (g) between time points by the number of days in between for three time periods: birth to weaning (ADG-BW), first week post-inoculation (ADG-PI1), and second week post-inoculation (ADG-PI2).

### Statistical analyses

Disease-related traits (abscess, bacteria, resistance), growth traits (ADG-BW, ADG-PI1, ADG-PI2) and commercial selection traits (NBA, WW) were analyzed using the Reml method with ASReml 3.0 [18].

#### Statistical models

Each trait was analyzed using specific models, which were all sub-models of the following “global” linear mixed animal model:

1 $${\mathbf{y}} = {\mathbf{X}}{\varvec{\upbeta}} + {\mathbf{Zu}} + {\mathbf{Vm}} + {\mathbf{Wl}} + {\mathbf{Op}} + {\mathbf{Sb}} + {\varvec{\upvarepsilon}},$$

where ${\mathbf{y}}$ is a vector of phenotype measures (one of the 7 traits); ${\varvec{\upbeta}}$ is a vector of fixed effects; ${\mathbf{u}}$ is a vector of animal genetic random effects with $\sim N(0,{\mathbf{A}}\sigma_{u}^{2}$) where ${\mathbf{A}}$ is the pedigree-based relationship matrix, with line included as a genetic group; ${\mathbf{m}}$ is a vector of maternal genetic random effects with $\sim N(0,{\mathbf{A}}\sigma_{m}^{2}$); ${\mathbf{l}}$ is a vector of the litter random effects with $\sim N(0,{\mathbf{I}}_{{\mathbf{l}}} \sigma_{l}^{2}$), where ${\mathbf{I}}$ is the identity matrix of appropriate size; ${\mathbf{p}}$ is the vector of permanent environment random effects with $\sim N(0,{\mathbf{I}}_{{\mathbf{p}}} \sigma_{p}^{2}$); ${\mathbf{b}}$ is a vector of the combined random effects of batch, room, and cage (BRC) with $\sim N(0,{\mathbf{I}}_{{\mathbf{b}}} \sigma_{b}^{2}$); ${\mathbf{X}}$ is a known design matrix for fixed effects; ${\mathbf{Z}}$, ${\mathbf{V}}$, ${\mathbf{W}}$, ${\mathbf{O}}$ and ${\mathbf{S}}$ are known design matrices for random effects, i.e. animal genetic, maternal genetic, litter, permanent environment, and BRC, respectively; and ${\varvec{\upvarepsilon}}$ is a vector of residual errors with $\sim N(0,{\mathbf{I}}_{{\mathbf{e}}} \sigma_{e}^{2}$).

For the experimental population, the fixed effects tested for each trait were: gender (2 levels), gestation length (4 levels i.e. 4 gestation lengths: 30, 31, 32, and 33 days), batch (5 levels i.e. five birth months within a year), parity of dam (6 levels), and signs of ERE/digestive disorders (3 levels). The scores for the ERE/digestive disorders were defined as follows: “0” for rabbits without signs of disorders, “1” for rabbits that showed signs of digestive disorders but not confirmed as signs of ERE, and “2” for rabbits with signs of ERE. The random environmental effects in the model were BRC and litter, with 196 levels (5 rabbits per level) and 305 levels, respectively, which will be referred to as non-genetic common environment shared by rabbits of the same litter. On average, each litter included 3.21 rabbits (from 1 to 10). Fixed effects were considered significant and included in the final model if the P-value was less than 0.05. To test the significance of the random effects, log likelihood values obtained from ASReml were used to perform a likelihood ratio test in the statistical software R [19] and included if the resulting P-value was less than 0.05. The final models used to estimate heritability and correlations contained only significant fixed and random effects.

For the selection population, the fixed and random effects that are used routinely by the breeding companies were included in the models. The fixed effects for WW were a combined effect of farm-year-month of birth (24 levels), number of kits born alive (12 levels), litter size at weaning (10 levels), and parity of the dam (5 levels), and for NBA, the combined effects of farm-year-season of kitting (28 levels) and the parity-physiological status (lactating or not at insemination, 9 levels). The random environmental effects included in the model were a permanent environment effect (2253 levels) for NBA, which accounts for the permanent environment effect for repeated measurement of NBA on does, and a litter effect (1483 levels) for WW. On average, NBA was recorded on 2.9 litters per doe (range from 1 to 12), and WW was recorded on 4.7 rabbits in each litter (range from 1 to 16).

Once the fixed and random effects were selected for each trait and population, heritabilities and genetic correlations between commercial selection traits and growth or disease-related trait were estimated with a linear model using three-trait analyses, including WW and NBA measured on the selection population, and a growth or disease-related trait measured on the experimental population. Due to convergence issues, correlations between growth and disease-related traits measured in the experimental population were estimated using two-trait analyses.

The disease-related traits abscess, bacteria, and resistance were also analysed as binary traits [0/1] using a threshold model. Classification of the disease-related traits into different categories may not be fully correct, which could lead to biased estimates of heritability. For abscess (and bacteria) as binary traits, 0 was assigned to rabbits that did not show any abscesses (and bacteria), and 1 to rabbits that showed signs of abscesses (low to severe) and (bacteria). For resistance as a binary trait, 1 was assigned to rabbits survived until the end of the experiment and that did not show any abscess or bacteria, and 0 was assigned to all other rabbits. In the threshold model, the 0/1 phenotype of a rabbit is linked to the explanatory variables in model (1) by a probit link function. Fixed effects were considered significant and included in the model if their P-value was less than 0.05. Random effects that were significant (P-value < 0.05) in the linear mixed animal model for the corresponding trait were fitted as random effects in the final threshold models for that trait. The threshold model gives estimates of the heritability on the underlying scale $\left( {h_{und}^{2} } \right)$. For comparison purposes, these estimates were transformed to the observed scale [20] using $h_{obs}^{2} = h_{und}^{2} \frac{{z^{2} }}{{\left[ {p\left( {1 - p} \right)} \right]}}$, where $p$ is the frequency of 1 s for the binary trait; $z$ is the ordinate (height) of a standard normal curve for the threshold that corresponds to “$p$”. The standard error of heritability estimate on the underlying scale was also transformed to the observed scale [20].

## Results

Of the 953 experimental rabbits used in the analysis, 72 showed no signs of abscess, 79 showed no bacterial growth in tissue samples, and 71 were resistant to pasteurellosis (no abscess, no bacterial growth, and alive until the end of the experiment).

The mean of each trait with its standard deviation (SD) are in Table 5. Although it is preferable to include all the effects used to randomize animals, we included only the fixed effects that were significant at the 5% level in the final models (Table 6). We observed no significant effect of gender for any of the traits and ignoring the effect of gender had no impact on estimates of variances and covariances. A significant effect of batch was observed for all the disease-related and growth traits except for ADG-PI2. Gestation length and dam’s parity showed significant effects on ADG-BW. There was no significant effect of the dam’s parity on the disease-related traits, probably because of the high health status of these dams, which were not exposed to *P. multocida*.

**Table 5:** Number of rabbits, means and standard deviations (in parentheses) for disease-related, growth, and commercial selection traits

Type of trait	Trait	Number of rabbits	Number of litters	Mean (SD)	Range (min–max)
Disease related (binary)	Abscess	951		0.92 (0.26)	0–1
	Bacteria	951		0.91 (0.27)	
	Resistance	953		0.07 (0.26)	
Disease related (categorical)	Abscess	951		2.61 (1.02)	0–4
	Bacteria	951		1.36 (0.96)	
	Resistance	953		3.19 (0.94)	
Growth	ADG-BW	980		27.14 (4.27)	14.32–39.74
	ADG-PI1	902		2.93 (18.23)	− 44.67 to 75.25
	ADG-PI2	852		19.76 (17.89)	− 42.29 to 74.29
Selection	NBA	2253	7528	10.34 (3.49)	0–20
	WW	11,423		836.43 (231.64)	218–1665

*ADG* average daily weight gain (g/day), *BW* from birth to weaning pre-inoculation, *PI1* during first week post-inoculation, *PI2* during second week post-inoculation, *NBA* number of kits born alive, *WW* weaning weight (g)

**Table 6:** Significant fixed effects for disease-related, growth, and commercial selection traits

Type of trait	Trait	ERE	Batch	Gestation length	Parity of the dam	Farm, year and month of birth	Number of kits born alive	Litter size at weaning	Farm, year and season at kitting	Parity and physiological status^a^
Disease related (binary)	Abscess	ns^b^	*	ns	ns	–	–	–	–	–
	Bacteria	ns	*	ns	ns	–	–	–	–	–
	Resistance	ns	*	ns	ns	–	–	–	–	–
Disease related (categorical)	Abscess	**	*	ns	ns	–	–	–	–	–
	Bacteria	**	*	ns	ns	–	–	–	–	–
	Resistance	**	**	ns	ns	–	–	–	–	–
Growth	ADG-BW	ns	**	**	**	–	–	–	–	–
	ADG-PI1	*	**	ns	ns	–	–	–	–	–
	ADG-PI2	*	ns	ns	ns	–	–	–	–	–
Selection	NBA	–	–	–	–	–	–	–	**	**
	WW	–	–	–	**	**	**	**	–	–

*ADG* average daily weight gain (g/day), *BW* from birth to weaning pre-inoculation, *PI1* during first week post-inoculation, *PI2* during second week post-inoculation, *NBA* number of kits born alive, *WW* weaning weight (g), – not tested

^a^Lactating or not at insemination

^b^** P-value < 0.001, * P-value < 0.05, ns: Not significant (P-value > 0.05)

ERE/digestive disorders showed a significant effect for all disease-related and growth traits but not for binary disease-related traits, and for ADG-BW (Table 6). In comparison to rabbits without a ERE/digestive disorder (score 0), rabbits that were diagnosed with a ERE/digestive disorder (scores 1 and 2) showed more severe signs of abscess (+ 0.69 ± 0.17 versus + 0.92 ± 0.17), more signs of bacterial growth (+ 1.13 ± 0.17 versus + 1.02 ± 0.16), and less resistance to pasteurellosis (− 1.04 ± 0.15 versus − 1.30 ± 0.15). This shows a significant interaction between pasteurellosis and ERE/digestive disorders, likely because ERE and digestive disorders are aggravating factors for pasteurellosis. For growth traits, compared to rabbits without a ERE/digestive disorder (score 0), rabbits diagnosed with a ERE/digestive disorder (scores 1 and 2) had lower ADG-PI1 (− 8.34 ± 3.42 versus − 9.67 ± 3.39 g/days) and ADG-PI2 (− 7.52 ± 4.37 versus − 9.89 ± 4.50 g/days).

### Heritabilities and correlations

Estimates of common litter and permanent environment effects, and of heritabilities are in Tables 7 and 8, respectively. For disease-related traits, heritability estimates ranged from 0.07 (± 0.04) to 0.16 (± 0.06) when based on the linear model, and from 0.16 (± 0.11) to 0.20 (± 0.08) when based on the threshold model, on the underlying scale. For growth traits, heritability estimates ranged from 0.11 (± 0.10) to 0.29 (± 0.07). For the commercial selection traits, heritability estimates were 0.33 (± 0.06) for WW and 0.05 (± 0.02) for NBA. Litter showed a significant effect for abscess on the linear scale, and for ADG-BW and WW. The permanent environment effect was significant for NBA. BRC and maternal genetic effects were not significant for any trait.

**Table 7:** Estimates of litter effects and of heritabilities (standard errors in parenthesis) for the disease-related traits^a^ analyzed with a threshold model

	Litter effect	Heritability	
		Underlying^b^	Observed^c^
Abscess	0.04 (0.09)	0.16 (0.11)	0.05 (0.03)
Bacteria	–	0.19 (0.07)	0.05 (0.02)
Resistance	–	0.20 (0.08)	0.06 (0.02)

^a^Expressed as binary variables [0–1]

^b^Underlying observed heritability estimates and standard error

^c^Observed heritability estimates and standard error

**Table 8:** Estimates of litter effect, permanent environment effect, and of heritabilities (standard errors in parenthesis) for the traits^a^ analyzed with a linear mixed model)

Type of trait	Trait	Litter effect	Permanent environment effect	Heritability
Disease-related^b^	Abscess	0.07 (0.04)	–	0.13 (0.07)
	Bacteria	–	–	0.08 (0.05)
	Resistance	–	–	0.16 (0.06)
Growth	ADG-BW	0.47 (0.05)	–	0.11 (0.10)
	ADG-PI1	–	–	0.29 (0.07)
	ADG-PI2	–	–	0.20 (0.06)
Selection	WW	0.23 (0.02)	–	0.33 (0.06)
	NBA	–	0.10 (0.02)	0.05(0.02)

^a^*ADG* average daily weight gain (g/day), *BW* from birth to weaning pre-inoculation, *PI1* during first week post-inoculation, *PI2* during second week post-inoculation, *NBA* number of kits born alive, *WW* weaning weight (g)

^b^Expressed as a categorical variable with five levels [0–4]

Estimates of the correlations between traits are in Table 9. Disease-related traits were analyzed on the linear scale to estimate these correlations. Disease-related traits displayed high estimated genetic and phenotypic correlations with each other. Abscess and bacteria showed a genetic correlation of 0.99 (± 0.16) and a phenotypic correlation of 0.58 (± 0.02). Resistance showed strong negative genetic correlations with abscess (− 0.99 ± 0.05) and bacteria (− 0.98 ± 0.07). In comparison to the genetic correlations, the phenotypic correlations of resistance with abscess (− 0.80 ± 0.01) and bacteria (− 0.84 ± 0.01) were slightly lower. Estimates of genetic correlations of disease-related traits with ADG-BW had large standard errors and should be interpreted with caution. The corresponding phenotypic correlations ranged from − 0.13 (± 0.03) to 0.12 (± 0.03). Estimates of genetic correlations of disease-related traits with growth traits for the first week (ADG-PI1) and second week (ADG-PI2) after inoculation were strong, with absolute values ranging from 0.70 (± 0.14) to 0.98 (± 0.09). The corresponding phenotypic correlations were moderately high, between 0.50 (± 0.02) and 0.68 (± 0.01) (absolute value). Estimates of genetic correlations among growth traits were inconclusive because of high standard errors, except a moderate genetic correlation of 0.52 ± 0.16 between ADG-PI1 and ADG-PI2. The phenotypic correlations between growth traits were low, except between ADG-PI1 and ADG-PI2 (0.32 ± 0.03). Estimates of genetic and phenotypic correlations of disease-related traits and growth traits with the commercial selection traits were not significantly different from 0.

**Table 9:** Estimates of genetic (above diagonal) and phenotypic (below diagonal) correlations (standard errors in parentheses) between disease-related, growth and commercial selection traits^a^

	Abscess	Bacteria	Resistance	ADG-BW	ADG-PI1	ADG-PI2	WW	NBA
Abscess		0.99 (0.16)	− 0.99 (0.05)	− 0.87 (0.42)	− 0.98 (0.09)	− 0.82 (0.14)	− 0.48 (0.33)	0.07 (0.38)
Bacteria	0.58 (0.02)		− 0.98 (0.07)	− 0.60 (0.42)	− 0.94 (0.17)	− 0.75 (0.17)	− 0.39 (0.36)	− 0.12 (0.41)
Resistance	− 0.80 (0.01)	− 0.84 (0.01)		0.75 (0.34)	0.98 (0.06)	0.70 (0.14)	0.42 (0.29)	− 0.07 (0.34)
ADG-BW	− 0.13 (0.03)	− 0.08 (0.03)	0.12 (0.03)		0.44 (0.30)	0.46 (0.34)	0.40 (0.45)	− 0.70 (0.56)
ADG-PI1	− 0.67 (0.02)	− 0.50 (0.02)	0.68 (0.01)	0.05 (0.03)		0.52 (0.16)	0.44 (0.23)	− 0.06 (0.29)
ADG-PI2	− 0.53 (0.02)	− 0.57 (0.02)	0.56 (0.02)	0.02 (0.03)	0.32 (0.03)		0.43 (0.27)	− 0.32 (0.31)
WW	− 0.10 (0.07)	− 0.07 (0.06)	0.10 (0.07)	0.07 (0.08)	0.14 (0.08)	0.11 (0.08)		0.36 (0.15)
NBA	0.01 (0.03)	− 0.01 (0.03)	− 0.01 (0.03)	− 0.05 (0.04)	− 0.01 (0.04)	− 0.03 (0.03)	0.06 (0.03)	

*ADG* average daily weight gain (g/day), *BW* from birth to weaning pre-inoculation, *PI1* during first week post-inoculation, *PI2* during second week post-inoculation, *NBA* number of kits born alive, *WW* weaning weight (g)

## Discussion

We investigated the potential of novel response traits as selection criteria for resistance to pasteurellosis, which to our knowledge, is the first study to investigate such diagnostic response traits. The systematic and detailed response trait measures on infected rabbits allowed us to detect genetic variation of resistance to pasteurellosis. The low to moderate estimates of genetic variance for these novel response traits, along with non-significant genetic correlations with the commercial selection traits, suggest that these response traits could be used as selection criteria for resistance to pasteurellosis.

### Effects included in the model

We investigated the contribution of random components such as maternal genetics, litter, and BRC to the phenotypic variance for analysis of disease-related traits on a linear scale and for growth traits. BRC and maternal genetic effects showed no significant contributions to the phenotypic variance. A non-significant contribution of the maternal genetic effects for a disease trait in rabbits has already been described by Eady et al. [13]. However, the same authors observed significant maternal genetic effects for a weight-related trait. The ratio of litter variance to phenotypic variance was only significant for abscess, ADG-BW, and WW. In the literature, similar estimates for common litter environment effects compared to our result for abscess (0.08 ± 0.04) were reported for a respiratory syndrome (0.057 ± 0.002) [12] and bacterial infections (0.046 ± 0.006 to 0.209 ± 0.017) [4, 13]. For ADG-BW, our estimate for this ratio (0.47 ± 0.05) was comparable to estimates obtained in previous studies (0.31 [21], and 0.40 [22]), in which litter effects were also investigated at early age intervals. However, low estimates of litter effects have also been reported for growth traits (0.11 [23], 0.22 [24], and 0.16 [25]). Such small litter effects have been observed for ADG traits that cover a later period of life in rabbits [24]. In addition to common environmental effects, litter effects include maternal environmental effects, which could be related to the milk that the mother passes to her kits [24], which suggests the importance of the suckling mother in weight gain. The magnitude of the litter effect reflects its importance from birth until weaning, and its inclusion in the model is expected to decrease the probability of estimating inflated heritabilities for growth traits.

### Heritability estimates for disease-related traits

Few studies have reported heritability estimates related to pasteurellosis resistance specifically in rabbits but genetic parameters for natural bacterial infections have been estimated in rabbits [4, 12, 13]. Pasteurellosis is one of the most common bacterial diseases in rabbits [3, 13] and usually manifests itself as a respiratory disease [3]. Hence, the estimates of heritability obtained here can be compared to estimates from these studies [4, 12, 13], as respiratory syndromes have been taken into account to define disease resistance in these studies.

Bacterial disease resistance assessed under field conditions was previously investigated in two commercial meat rabbit populations [13]. Rabbits were scored for infection based on clinical signs such as, but not limited to, respiratory problems, snuffles, and abscesses. The highest heritability estimates were 0.042 (± 0.012) based on the linear model (treating the trait as continuous), and 0.379 (± 0.106) on the underlying scale when using the threshold model. Estimates of genetic variability for infectious diseases in French rabbits included respiratory syndromes, among many other traits [12]. Respiratory syndromes were observed for 4% of the population, with a heritability estimate of 0.041 (± 0.004). Our heritability estimates for the response traits were higher than observed in the above-mentioned studies [4, 12, 13], which could be due to several reasons. First, in our study, the rabbit population was experimentally exposed to *P. multocida*, whereas in the previous studies field data were used, for which uneven exposure may lead to a low incidence of disease. Such a scenario was also observed in a study on ERE, in which heritability estimates of the disease decreased from 0.21 (± 0.16) for an experimental rabbit population [26], to 0.08 (± 0.02) in field data [27]. Second, the diagnostic approach in our study provided the “true *Pasteurella* infection” status of each rabbit, thus our estimates better reflect true genetic differences in resistance in the population. The healthy rabbits did not show any internal signs of pasteurellosis, which might not be the case for previous studies, as they took only external visible signs into account. Our heritability estimates of pasteurellosis resistance traits are similar to those reported for lung lesions in Spanish rabbit lines [from 0.07 (± 0.03) to 0.18 (± 0.09)], which could be due to the use of a similar diagnostic approach in both studies. In [11], fresh lung lobes were scored based on the extent of the lesions in the lungs of euthanized rabbits under natural *Pasteurella* infection.

Many infectious diseases, such as rabbit haemorrhagic disease, ERE, and myxomatosis exist in rabbit populations [3, 28]. However, genetic parameters for resistance to these diseases are scarce in the literature. Only Baselgea et al. [11] reported estimates of genetic parameters for pasteurellosis resistance in rabbits. Our heritability estimates follow a similar trend as those for ERE/digestive disorders in rabbits, which ranged from 0.05 (± 0.05) to 0.21 (± 0.16) in two studies [26, 27]. Based on these reports, this disease trait was included in the breeding program of the Hypharm breeder to reduce the prevalence of ERE. Furthermore, Garreau et al. [29] investigated the status of susceptibility to digestive disorders in experimental progenies from a rabbit population selected for resistance to digestive disorders and observed a significant reduction in mortality between resistant and susceptible experimental rabbits. Another study [30] on rabbits from the same line showed a reduction in clinical signs of disease by 0.12 genetic standard deviation per year between 2008 and 2016. This result suggests the potential of reducing the prevalence of pasteurellosis in rabbits by introducing pasteurellosis resistance traits in a breeding program.

We also estimated the genetic variance of the response traits in a binary form [0/1]. Incidences for the binary traits were outside the 10 to 90% range (Table 5), which might dissociate the assumption of independence between the mean and variance when estimating variance components on the linear scale [31]. Thus, we applied a threshold probit link function for the analysis of the binary traits. To simplify comparisons, heritability estimates on the underlying scale were transformed to the observed scale. Resulting estimates of heritability on the observed scale ranged from 0.05 (± 0.02) to 0.06 (± 0.02) and were much lower than those obtained from the linear model (Table 8). Greater genetic variation on the linear scale shows the advantage of taking the severity of pasteurellosis into account when recording response traits.

### Heritability estimates for commercial selection traits

Estimates of variance components for NBA and WW were consistent with data in the literature [32–34], which ranged from 0.04 to 0.16 for NBA and from 0.26 to 0.29 for WW.

### Heritability estimates for growth traits

#### Before inoculation

Heritability estimates obtained for ADG before inoculation (ADG-BW) were low, which could be explained by the use of data recorded at an early age interval. For rabbits at an early age, the effect of the maternal environment component is larger, which might reduce the effect of the rabbit itself [24]. We also observed a large significant effect of litter (0.47 ± 0.05) for ADG-BW. Such a pattern showing an increase in direct animal genetic effects (thus increasing heritability estimates) for ADG with increasing age of the animal was previously reported: low to moderate heritability estimates for ADG between 5/6 and 10 weeks of age (0.25 [35], 0.21 ± 0.01 [36], 0.23 ± 0.02 [25], 0.204 ± 0.008 [37], and 0.15-0.17 [38]) but higher estimates of heritability for ADG after 15 weeks of age (0.48 [23], and 0.29 [39]).

#### After inoculation

The heritability estimate for ADG during the first week post-inoculation (ADG-PI1) was moderate (0.29 ± 0.07) and similar to that for ADG during the second week post-inoculation (ADG-PI2) (0.20 ± 0.06). Most rabbits in our study showed a strong decrease in daily weight gain during the first week post-inoculation [from − 44.7 to 75.3; mean: 2.9 g/d (SD = 18.2)]. The reduced body weight gain in the first week post-inoculation appears to result directly from pasteurellosis infection. However, rabbits coped better during the second week post-inoculation, i.e. the mean of ADG-PI2 increased to 19.8 g/days (SD = 17.89) (from − 42.3 to 74.3). Thus, the heritability estimate of 0.29 for ADG-PI1 suggests that it could be advantageous to select rabbits against pasteurellosis resistance at an early phase of infection during which most of the genetic variability in ADG is observed.

Heritability estimates of the traits obtained from both univariate analyses and bivariate analyses (results not shown) were similar, which indicates that our models were robust. Because we used crossbred populations, heritabilities might be slightly overestimated. The effect of heterosis was not analyzed because of the lack of phenotypic information for the sires and dams.

### Correlation estimates

#### Among disease-related traits

Disease-related traits showed strong estimates of genetic correlations but comparatively lower phenotypic correlations between each other. All the rabbits with a severe abscess score did not always have a severe bacteria score or vice versa. Since the resistance score is a composite trait based on the abscess and bacteria scores, strong relationships were expected at both the genetic and phenotypic levels. The strong genetic correlation estimates suggest that rabbits that are genetically more susceptible to harbor many bacteria will also show severe abscesses. This indicates that any of the response traits evaluated here could be an effective indicator to improve disease resistance against pasteurellosis.

#### Between disease-related traits and growth traits

Estimates of genetic correlations between growth pre-inoculation (ADG-BW) and disease-related traits (abscess and bacteria) were negative and favorable but had large standard errors (Table 9). The estimate of the genetic correlation between ADG-BW and resistance was positive and favorable, which could result from the definition of the resistance trait. Thus, favorable genetic correlations suggest that breeding programs can combine selection for resistance to pasteurellosis and selection for growth. Previous studies in rabbits with natural infection reported low to moderate negative genetic correlations between disease-related traits and growth traits [12, 13, 27]. Estimates of phenotypic correlations between ADG-BW and disease-related traits were weaker than the genetic correlations (Table 9).

Post-inoculation, we observed strong negative genetic correlation estimates between disease-related traits (abscess and bacteria) and growth traits (ADG-PI1 and ADG-PI2) and strong positive genetic correlations between resistance and growth traits. These relationships suggest that rabbits that are genetically resistant to pasteurellosis have a better ADG post-inoculation than less resistant rabbits. Thus, ADG-PI1, measured over the first week post-inoculation could be a good indicator trait for pasteurellosis resistance since recording ADG-PI1 after inoculation is easier, cheaper, and faster than recording disease-related traits (abscess, bacteria, and resistance).

The standard bivariate analyses that we used did not take any underlying simultaneous or recursive causal effects that may exist between traits into account [40]. A better understanding of such underlying causal effects between traits would support better decision making in breeding programs [41]. Various structural equation models have been suggested that investigate causal effects of genetic correlations [39, 40] but they have rarely been applied to infer genetic causal relationships between traits [41, 42] and should be explored in the future.

#### Between disease-related, growth, and commercial selection traits

Moderate and favorable genetic correlations between resistance to infectious diseases and WW (− 0.34 ± 0.12) were reported in [34]. In this study [34] and in [11], estimates of genetic correlations of resistance to infectious diseases or respiratory diseases with production traits such as NBA or live weight at market age were not significantly different from zero. Our results suggest a favorable genetic correlation between resistance and WW and an unfavorable genetic correlation between resistance and NBA (Table 9). However, the number of inoculated rabbits was not large enough to allow accurate estimation of genetic correlations (high standard errors) and thus to draw definite conclusions.

### Implications for breeding programs

Our results show that the host genetics contributes to differences in resistance to pasteurellosis in rabbits. The remaining question is how to integrate these useful findings in a breeding scheme. Various options are possible, depending on the financial resources available. The easiest but probably less efficient option would be to keep selecting on weaning weight or growth rate before weaning to get an indirect correlated response on resistance to pasteurellosis. However, the high standard errors on the genetic correlations between resistance and ADG-BW or WW do not provide high confidence on the magnitude of this correlated response. A more efficient (but more costly) option would be to repeat this experimental infection trial with *P. multocida* on sibs of the selection candidates in each generation. Growth rate in the first week after inoculation (ADG-PI1) may be the most promising trait to select on because it had the highest heritability (0.29 ± 0.07) among the traits measured post-inoculation, and it had a very high estimated genetic correlation with resistance (0.98 ± 0.06). This trait is also easier to measure than the other response traits because no laboratory analyses or post-mortem examinations are required, and the duration of the challenge can be reduced to 1 week.

However, performing repeated disease challenges raises ethical issues, and an adjustment of the challenge may be needed to decrease the intensity of the clinical signs, by reducing the inoculation dose or by changing the *P. multocida* strain. Alternatively, future research could focus on the detection of blood markers or on the development of in vitro immune response tests that could predict response to pasteurellosis without experimental challenge.

Another approach that should be explored is the use of genetic markers. DNA samples from the rabbits used in this study and of their parents have been preserved and will be used for genotyping. If a limited number of genes or quantitative trait loci control resistance to pasteurellosis, gene or marker-assisted selection [43] could be proposed. If resistance is a polygenic trait, genomic prediction [43] should be considered. The main drawback would be the cost of genotyping and performing regular experimental challenges on sibs of the selection candidates to ensure sufficiently high prediction accuracy. To date, genomic prediction has not been implemented in rabbits, mainly for reasons summarized in [44, 45], i.e. the cost of genotyping is high compared to the individual animal value, rabbit breeding programs have a pyramidal structure, with selection in pure lines for performance expressed in crossbred animals, rabbits have a short generation interval, and several logistical issues. Thus, further economic studies are needed to compare strategies that could be used to incorporate selection for resistance to pasteurellosis in the breeding schemes.

## Conclusions

Genetic parameters for novel resistance traits to experimental infection *P. multocida* were evaluated. Results provide evidence for a genetic basis for these response traits in this experimental crossbred rabbit population and for the potential to decrease the prevalence of pasteurellosis by selecting resistant rabbits on any of the response traits evaluated. Strong favorable estimates of genetic correlations between disease response traits and growth traits post-inoculation suggest that growth after inoculation could be an alternative efficient indicator trait for pasteurellosis resistance. However, implementing the recording of these traits under industrial conditions is not easy. Instead, these traits could be used to facilitate the detection of markers of pasteurellosis resistance in rabbit populations. The moderate heritability of the disease response traits and their non-significant genetic correlations with the commercial selection traits suggest that a breeding program could combine selection for resistance to pasteurellosis and selection for growth rate without infection.

## Data Availability

The datasets analyzed for the current study are not publicly available because they are the property of private companies but are available from the corresponding author on reasonable request.
